# Mini-canaloplasty as a modified technique for the surgical treatment of open-angle glaucoma

**DOI:** 10.1038/s41598-020-69261-y

**Published:** 2020-07-30

**Authors:** Marek Rękas, Joanna Konopińska, Anna Byszewska, Zofia Mariak

**Affiliations:** 1 Department of Ophthalmology, Medical University of Białystok, M. Sklodowska-Curie 24A STR, 15-276 Białystok, Poland; 2 Department of Ophthalmology, Military Institute of Medicine, Szaserów 128, 04-141 Warsaw, Poland

**Keywords:** Clinical trial design, Health care, Medical research

## Abstract

Authors present a modified surgical technique for canaloplasty without preparing the classical trabeculo-Descemet’s membrane (TDM) and having to close sutures. Twelve patients with open-angle glaucoma (OAG) (aged 58–77 years) received the modified technique, which does not require the deep scleral flap to be excised, an intrascleral lake to be created, or TDM dissection. After accessing the Schlemm’s canal (SC), cannulation and placement of the sutures are made similar to those in the classical canaloplasty. The conjunctiva is closed via bipolar diathermy. The mean intraocular pressure (IOP) before surgery was 18.0 ± 8 mmHg, and the mean number of anti-glaucoma medications taken was 3 ± 1. Mean IOP at the end of the observation period (18.0 ± 6.0 months) was reduced by 23% (15.5 ± 4.1 mmHg), while the mean number of medications taken was reduced to 0.25 ± 1.0. In all eyes, the SC was successively opened, with no cheese-wiring. Adverse events included microhyphaema, mild corneal oedema, and folds in the TDM. The eyes recovered spontaneously within a few days after the procedure. The mini-canaloplasty technique may reduce the risk of complications associated with classical canaloplasty while effectively lowering the IOP in patients with OAG.

## Introduction

The gold standard surgical treatment for glaucoma is currently trabeculectomy, but the prevalence of canaloplasty is increasing. Since its introduction almost 15 years ago, many thousands of these procedures have been performed worldwide^1–4^. Classified as a non-penetrating anti-glaucoma procedure alongside deep sclerectomy, it enables the reduction of intraocular pressure (IOP) while avoiding many clinically significant complications related to the formation of a filtering bleb in fistular procedures. It is favourable for patients with a high risk of hypotony, scarring, and inflammation. The procedure also ensures safety and minimal interruption in the eye’s structure, which results in quicker recovery and less burden for the patients^4^. However, it also has limitations; the preparation of the classical trabeculo-Descemet’s membrane (TDM) in the clear cornea is technically challenging and bears the risk of a TDM rupture with iris prolapse. Due to the technical difficulty of non-penetrating anti-glaucoma operations, procedures are continuously being modified to prevent such complications and facilitate the surgical technique^5–9^. Therefore, this study aimed to present a modified, mini-incision technique for accessing the Schlemm’s canal (SC) with the intention of decreasing the risk of surgical problems while effectively lowering IOP. The hypotensive effect will only be induced by the reinforcement of wall tension in the SC and facilitation of the conventional aqueous drainage pathway from the anterior chamber through the SC, without creating an intrascleral lake. We describe this modified technique and the preliminary results of 12 cases to support the proposed method.

## Results

The mini-canaloplasty technique was performed in 12 patients (aged 58–77 years; mean age 67.2 ± 5). The mean IOP before the procedure was 18.0 ± 8 mmHg, and the patients were on an average of 3 ± 1 anti-glaucoma medications. The mean pressure at the end of the observation period (18.0 ± 6.0 months) was reduced by 23% (15.5 ± 4.1; range 10–19 mmHg), and the mean number of medications decreased to 0.25 ± 1.0. The mean preoperative corrected distance visual acuity (CDVA) was 0.6 ± 0.2, and this improved to 0.3 ± 0.2 post-surgery (Table 1).

**Table 1:** Patients’ demographic data and post-surgery results.

Demographic characteristics		
Age	67.2 ± 5	
Gender (female/male)	8/4	
Glaucoma type (POAG/NTG/PGX)	7/3/2	
Follow-up (months)	18.0 ± 6.0	

	Before surgery	Post-surgery
IOP (mmHg, mean ± SD)	18.0 ± 8	15.5 ± 4.1
CDVA (logMar, mean ± SD)	0.6 ± 0.2	0.3 ± 0.2
Number of anti-glaucoma medications (range ± SD)	3 ± 1	0.25 ± 1.0

*CDVA* corrected distance visual acuity, *IOP* intraocular pressure, *logMAR* logarithm of the minimal angle of resolution, *POAG* primary open-angle glaucoma, *NTG* normal tension glaucoma, *PGX* pseudoexfoliative glaucoma, *SD* standard deviation.

In five cases, a hyphaema was observed in the anterior chamber on the first day after surgery, but it spontaneously resolved within 7 days. On the first day after the procedure, three patients had mild corneal oedema and folds in the TDM; however, these spontaneously resolved within 7 days after the surgery. Macular oedema was observed in one patient within the first 30 days after the surgery and this was resolved through the use of topical treatment with steroids and non-steroid anti-inflammatory medications.

## Discussion

In this case series, mini-canaloplasty was a safe and effective surgical procedure; no ruptures of the TDM with iris prolapse were observed and only minor post-surgery complications occured. In the classical technique where both the superficial and deep scleral flap are dissected, the second flap is opened very near to the choroid to locate the SC. The TDM, through which aqueous solution is filtered, has a thickness of 0.25 ± 0.09 mm and should remain undisturbed during the classical canaloplasty or deep sclerectomy after the removal of the roof above the SC. According to Riva et al*.*, the TDM is perforated in as many as 8.5% of cases during the dissection of the anterior part of the deep flap in the clear cornea, particularly over the course of a learning curve^10^. While the classical canaloplasty is considered to be a low-traumatic method for treating glaucoma, our modified method allows for even less interference in the eye’s structure. A smaller scleral flap means that there is essentially no risk of TDM damage. The absence of a deep flap eliminates the possibility of scleral perforation during dissection.

Some authors stress that a filtering bleb is also formed, and subconjunctival drainage occurs in 2.5–10% of patients after the classical canaloplasty^11–14^. Well-documented studies have reported bleb-related endophthalmitis after a trabeculectomy in up to 9.6% cases, a number that increases a further 2.5% per patient-year with the addition of mitomycin C^15,16^. To date, no cases of endophthalmitis that could be attributed to canaloplasty have been reported. In our case series, there was no filtration bleb in any of the patients after the surgery.

The absence of a classical deep scleral flap eliminates the possibility of scleral perforation during dissection. However, this requires intimate knowledge of the sclera, SC, and corneal limbus anatomy by the surgeon. The cut of a small block of tissue must be directly aimed above the SC, which is approximately 2–2.5 mm behind the limbus^17^. During the conventional preparation, a useful landmark for the surgeon is the white sclera tissue of the spur, which can be visualised. This landmark cannot be visualised during mini-canaloplasty. Hence, the technique may be more challenging and have a lower success rate, especially during the learning curve.

Sutureless closure of the post-operative wound leads to faster and easier convalescence as well as a lack of post-operative astigmatism. We assume that lesser tissue trauma will allow for earlier withdrawal of anti-inflammatory medications during the post-operative period. Moreover, the need for additional laser goniopuncture after the procedure is eliminated. The positive influence of this operation on the patient’s quality of life over traditional anti-glaucoma procedures may be similar to microinvasive glaucoma surgery or phacoemulsification.

Complications related to blood reflux from the SC or intensification of bleeding into the anterior chamber are among the undesirable effects of the method that we have presented. These are short-term complications and generally do not require additional intervention. Furthermore, blood reflux is considered to be a beneficial prognostic factor in the case of canaloplasty^18^.

Mini-canaloplasty can be targeted to the same population of patients as the conventional method because the patient selection criteria are the same for both procedures. Moreover, the same possible complications can occur as in the classical canaloplasty, which include hyphaema^18^, no possibility of passing a catheter through the SC as a result of external obstruction by blood or fibrin^3^, and an incorrect route of passage^2,3^. The success rate of canal cannulation is reported to range from 74^2^ to 89.9%^4^. Difficulties with intubation may occur in the event of unforeseen anomalies in the structure of SC as well as after prior laser procedures on the filtration angle (argon laser trabeculoplasty) causing destruction of the trabecular meshwork’s structure and excessive scarring^19^. In our case series, we did not observe these complications. Another serious complication that may occur is haemorrhagic Descemet membrane detachment—and although techniques are described in the literature for managing such cases, this is a potentially sight-threatening complication^20,21^. We did not observe this complication among our 12 cases. Mini-canaloplasty as well as classical canaloplasty have major advantages over trabeculectomy including a better safety profile, faster convalescence after surgery, and less post-operative control visits owing to the lack of need of additional procedures such as needling, subconjunctival injections of antifibrotic agents (mitomycin or 5-fluorouracyl) and removal of adjustable sutures.

This modification can be applied not only as a first-choice procedure but also in cases where obstruction of SC is found during classical canaloplasty. The performance of this type of modification above the site of obstruction enables control of the site and facilitates further intubation of SC. In addition, this operation may be performed in every quadrant as long as the canal’s walls remain intact and it is anatomically possible to allow intubation. A low number of manipulations makes this procedure easier to perform in quadrants where access is more difficult and the surgeon’s manoeuvres are hindered. Performance of this procedure in lower quadrants as a first-choice procedure would make it possible to conserve upper quadrants, with traditional procedures being performed later if necessary. A smaller superficial scleral flap requires a less mobile area of the conjunctiva, and in the case of another procedure on the same eye in the future, this could be rationally justified. There are also studies describing the performance of canaloplasty after an unsuccessful trabeculectomy procedure^22^. If the continuity of the SC is preserved, performing mini-canaloplasty appears to be beneficial in such cases.

The efficacy of the mini-canaloplasty technique and extent to which it can reduce the IOP are still basic concerns. As Mastropasqua et al*.* assumes, transscleral drainage plays a large role in the hypotensive mechanisms of canaloplasty^12^. In this technique, IOP reduction depended purely on the conventional outflow through the SC and collector channels because no intrascleral lake was cut and therefore, no filtering bleb was formed. Because the role of individual components that could have an effect on the hypotensive components of canaloplasty are discussed in the literature, this fact sheds new light on the role of intubation of the SC in IOP regulation^4^. After performing approximately a dozen operations of this type and observing them over several months, we have found their hypotensive action is similar to that of classical canaloplasty.

A prospective, randomised clinical trial is currently being conducted to assess three different canaloplasty procedures, including mini-canaloplasty. The trial is registered at https://www.clinicaltrials.gov (identifier NCT02908633). A comparison of these surgical methods may provide an answer to whether improved drainage via the conventional pathway is solely responsible for the hypotensive effect and extent to which the intrascleral lake is involved in the traditional canaloplasty*.*

The limitations of this paper include the small sample size, unknown role of the intrascleral lake in terms of IOP reduction in the canaloplasty, and the short observation period. Certainly, the presented technique requires the expertise of skilled surgeons to be performed successfully. It is intended for the surgeons who are proficient in classical canaloplasty and ready to move to the next step because of the difficulty in finding the canal.

In conclusion, modern surgeries are focused on minimising injuries and instrumentation, as well as shortening surgery time (i.e. ab interno canaloplasty^23^ or flap-sparing canaloplasty^17^). The mini-canaloplasty technique is a non-penetrating procedure that attempts to perform canaloplasty without simultaneous deep sclerectomy and cutting out deeper layers under the superficial scleral flap. Thus certain complications related to non-penetrating deep sclerectomy can be avoided. Moreover, the main advantages of this modified method are shorter time of surgery, less traumatisation of tissues, and no need for suturing the sclera and conjunctiva. In our opinion, this procedure takes less time in experienced hands, but the learning curve may be longer than in classical canaloplasty.

## Methods

This study was conducted in the Department of Ophthalmology, Military Institute of Medicine, Warsaw, Poland. The project is compliant with the principles of the International Helsinki Federation for Human Rights and is aligned with the Good Clinical Practice for Trials on Medicinal Products developed by the European Union. It has been approved by the Bioethics Commission of the Military Institute of Medicine in Warsaw as it was in the previous studies^24–26^.

The protocol of the examination was similar to those we previously employed^24–26^. The indications for the procedure were the same as those of the conventional method: patients with primary open-angle glaucoma, pseudoexfoliative glaucoma, and normal tension glaucoma and in which a satisfactory IOP level was not achieved despite maximum tolerable hypotensive treatment (both topical and systemic). Additional inclusion criteria were as follows: documented progression of loss of field of vision, significant daily IOP fluctuations, no cooperation of the patient with application of anti-glaucoma treatment, and allergy to topical medications. Written informed consent for performing this modified procedure was obtained from all patients after they had been informed of the nature of the procedure and other surgical alternatives. Exclusion criteria were as follows: consent not provided to participate in the study, prior surgical and laser procedures in the area of the eye, narrow or closed-angle glaucoma, post-inflammatory or post-traumatic secondary glaucoma, chronic illness of the cornea or optic nerve, advanced macular degeneration, active inflammatory process, pregnancy, and systemic steroid therapy^24–26^.

### Preoperative examination

Detailed data about previous treatments and surgical procedures were collected from the patients at the time of qualification. Before surgical treatment, all patients underwent a basic examination, which included determination of the IOP, uncorrected distance visual acuity (UDVA), CDVA, and ophthalmologic examination of the anterior and posterior segments of the eye. If a patient had a coexisting cataract, then the intraocular lens was measured in order to have combined phaco-canaloplasty procedure^24–26^. In three cases where a concomitant cataract was found, combined canaloplasty and phacoemulsification procedure was performed. In these cases, the power of the artificial intraocular lens was calculated using the SRK/T regression formula on the IOL Master 700 (Carl Zeiss Meditec). The cataract surgery was conducted first, followed by the anti-glaucoma procedure.

### Surgical technique

**Figure 1:**
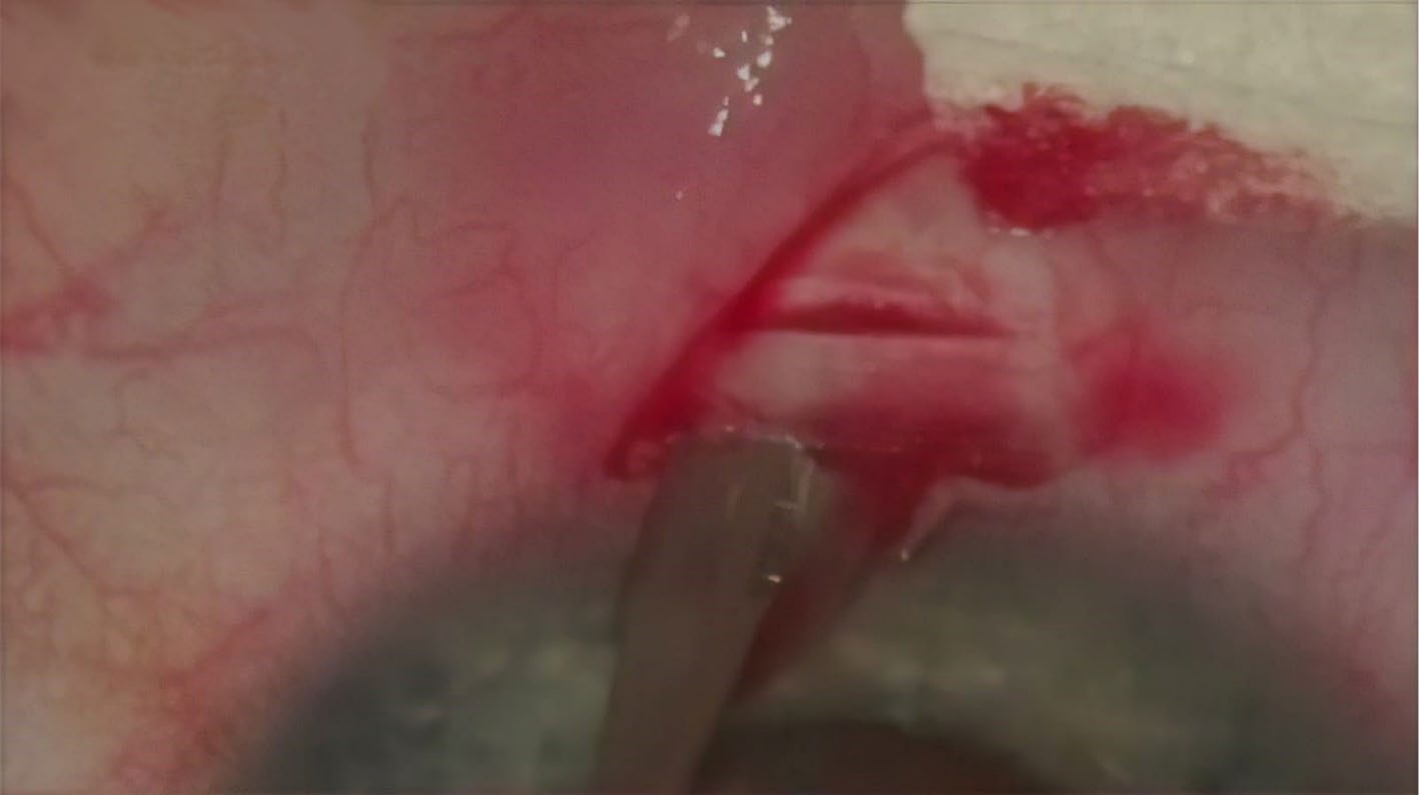
Opened conjunctiva; 4 × 1 mm superficial scleral flap constructed.

After anaesthesia with 2% xylocaine, a 4-0 suture was placed on the superior rectus muscle extraocularly, and a speculum was applied to the eyelids^27^. In the first stage of the procedure, the ocular conjunctiva was cut off from the limbus, and the vascular bed of the episclera was scraped off over an area of approximately 5 mm^2^. In the next stage, two parallel cuts of the sclera were performed, one in the limbus and the other 2 mm from the limbus, enabling dissection of a 4 × 1.5 mm superficial flap of a thickness corresponding to one-third of the sclera without cutting away one of the flap’s side walls^27^ (Fig. 1).

**Figure 2:**
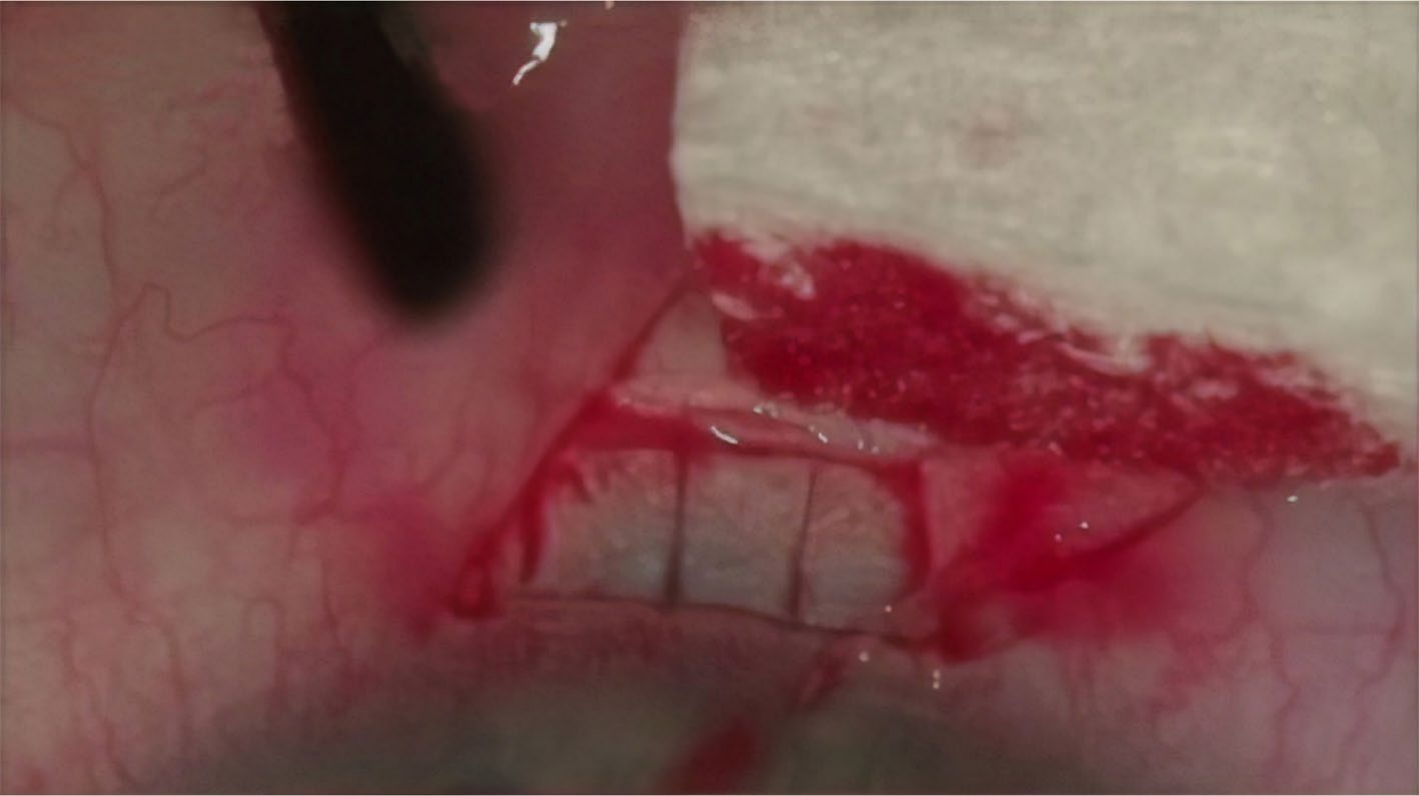
Deep scleral flap measuring 1.5 × 1. 5 mm constructed.

**Figure 3:**
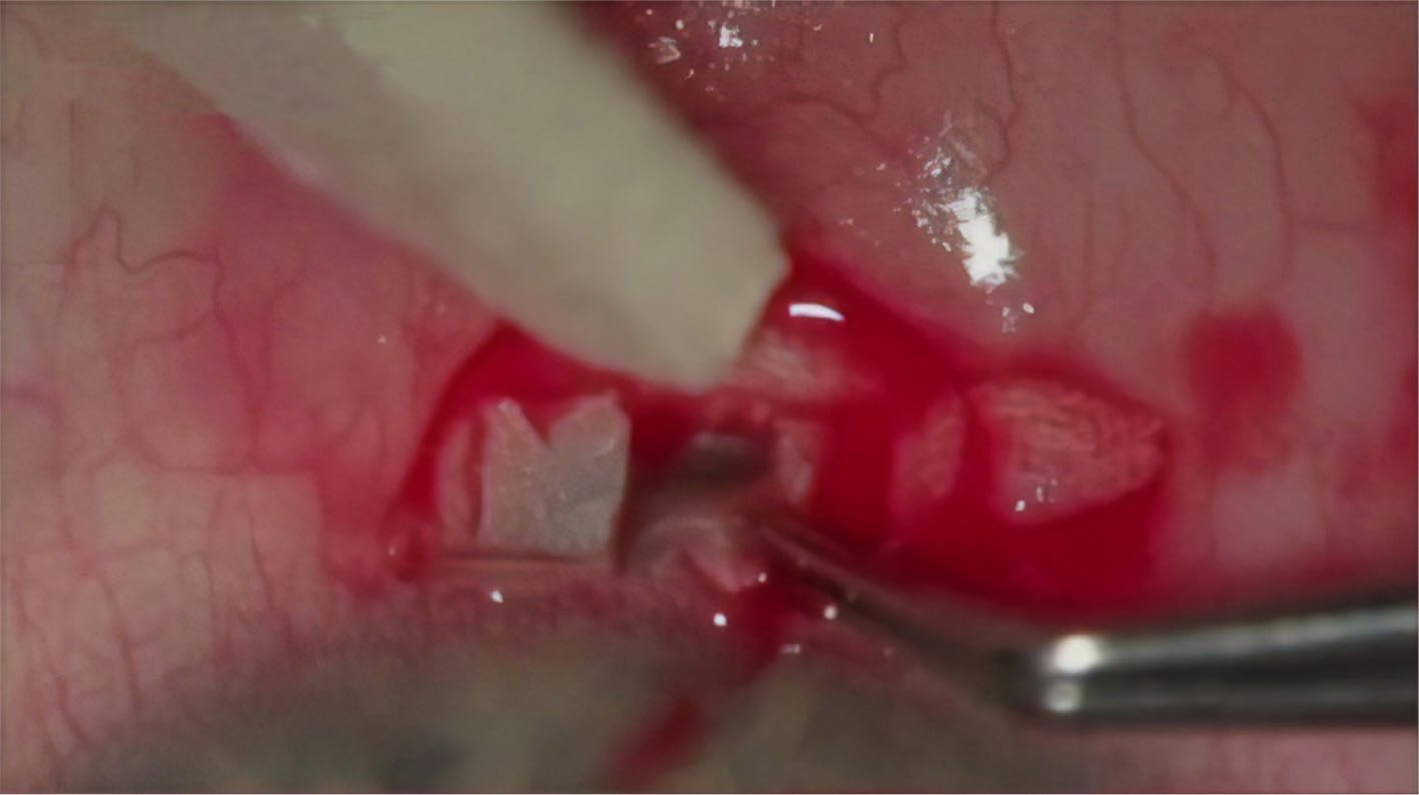
Deep scleral flap removed, under which the trabeculo-Descemet’s membrane is observed.

A 1.0 × 1.0 mm scleral block with a pedicle from the side of the cornea was cut out under the flap, directly above the SC. The perpendicular cuts in the sclera revealed the scleral spur, inlets into SC, and a small area of the TDM^27^ (Figs. 2, 3).

**Figure 4:**
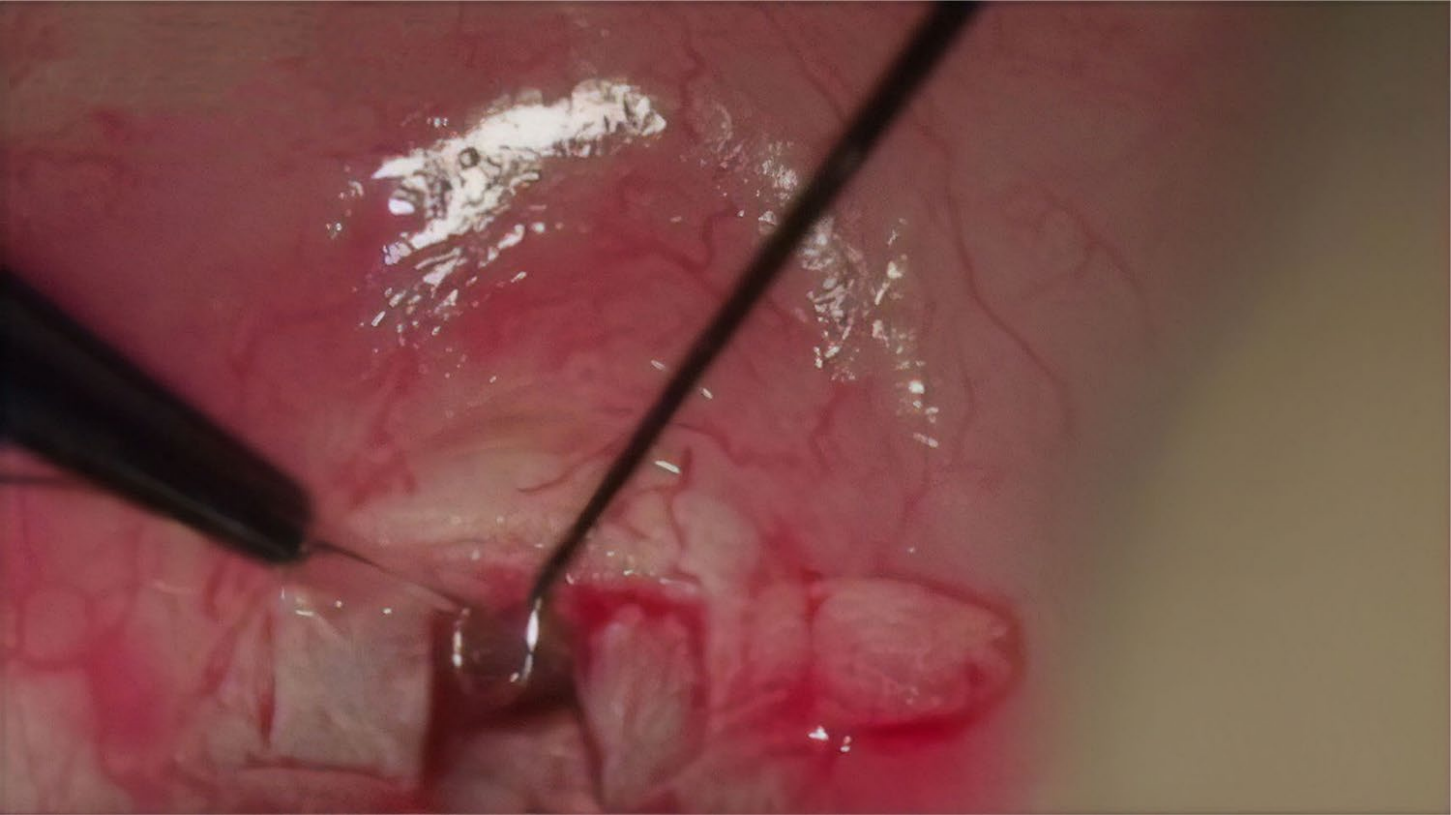
Catheter placed in both ostia of the Schlemm’s canal following successful catheterisation of the canal.

The next stages of the procedure were the same as in a standard canaloplasty. A catheter with a diameter of 250 µm (iTrack250A, Ellex) was inserted into the lumen of SC, and the canal was intubated throughout its entire circumference. Insertion of the catheter through a 1.0 × 1.0 mm window required its tip to be levered by means of an additional instrument. In our case, we used a micro-hook^27^ (Fig. 4).

**Figure 5:**
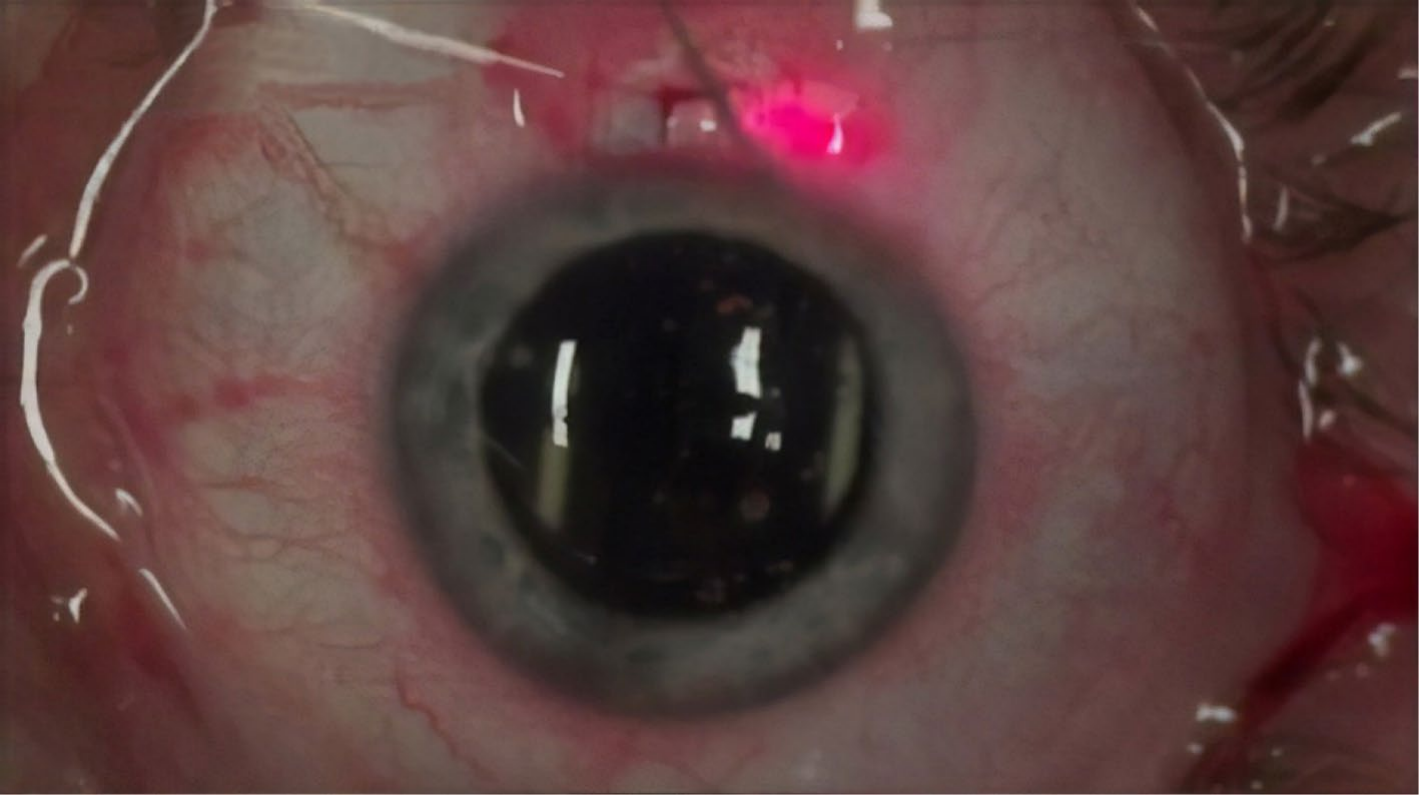
Viscodilation and insertion of suture into the canal completed.

A 10-0 Prolene suture was tied to the catheter, then pulled into the canal while administering 1.4% sodium hyaluronate (Healon GV) every 30° by one-eighth rotation of the syringe’s tip. After the suture was cut away from the catheter, a loop was made to control the suture’s tension in the canal. The loop was tightened until the curvature of the corneal limbus was moulded^27^ (Fig. 5).

**Figure 6:**
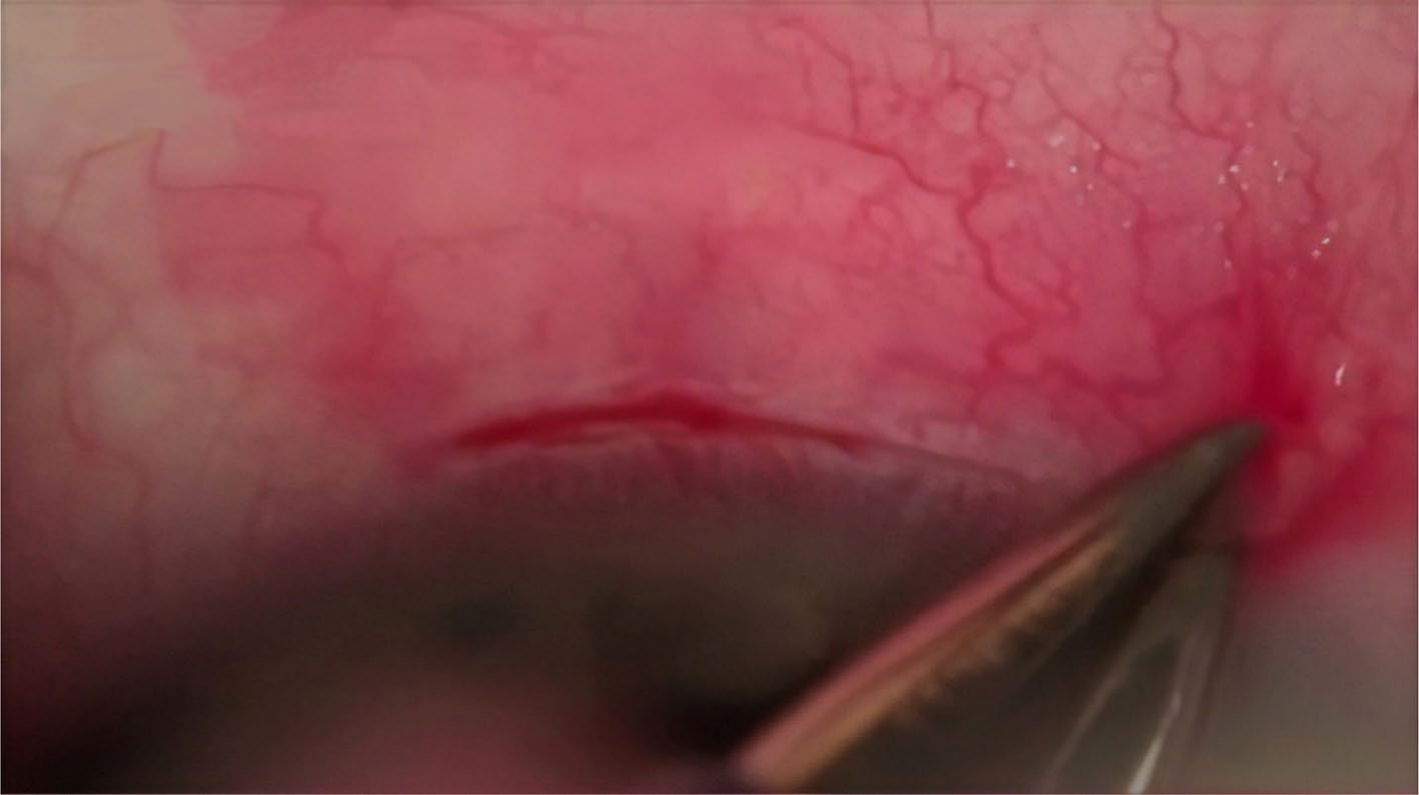
Closure of conjunctiva with diathermy requiring no suture.

The window was closed with the scleral block and loosely covered with the superficial scleral flap. The conjunctiva was closed via bipolar diathermy at the end of the procedure^27^ (Fig. 6, Supplementary Video S1).

### Post-operative protocol

During the follow-up visits, the IOP was measured using the Goldmann applanation tonometer, whereas the CDVA was measured using a logarithm of the minimal angle of resolution (logMAR) + notification. All IOP measurements were taken between 8 and 10 a.m.^24–26^. The anterior chamber and retina were assessed. During post-operative assessments, the complications and the number of anti-glaucoma medications were recorded^24–26^.

No anti-glaucoma medications were allowed on the day of the operation. When the operation did not produce the expected results, the medications were re-administered as recommended by the European Glaucoma Society^24–26^.

## Supplementary information

Supplementary Information.

Supplementary Video S1.

## Data Availability

All materials and information are available upon an e-mail request to the corresponding author. Names and exact data of the participants of the study may not be available because of privacy policies.

## References

[1] Stegmann RC (1995). Visco-canalostomy: A new surgical technique for open angle glaucoma. An. Inst. Barraquer..

[2] Lewis RA (2009). Canaloplasty: Circumferential viscodilation and tensioning of Schlemm canal using a flexible microcatheter for the treatment of open-angle glaucoma in adults: 2-year interim clinical study results. J. Cataract Refract. Surg..

[3] Grieshaber MC, Pienaar A, Olivier J, Stegmann R (2010). Clinical evaluation of the aqueous outflow system in primary open-angle glaucoma for canaloplasty. Invest. Ophthalmol. Vis. Sci..

[4] Roy S, Mermoud A (2006). How does nonpenetrating surgery work?. J. Fr. Ophtalmol..

[5] Seuthe AM (2016). Modified canaloplasty with suprachoroidal drainage versus conventional canaloplasty-1-year results. Graefes Arch. Clin. Exp. Ophthalmol..

[6] Xin C, Chen X, Shi Y, Wang H, Wang N (2016). Modified canaloplasty: A new, effective, and safe option for glaucoma patients with a disrupted Schlemm canal wall. J. Glaucoma..

[7] Szurman P, Januschowski K, Boden KT, Szurman GB (2016). A modified scleral dissection technique with suprachoroidal drainage for canaloplasty. Graefes Arch. Clin. Exp. Ophthalmol..

[8] Körber N (2017). Canaloplasty ab interno—A minimally invasive alternative. Klin. Monbl. Augenheilkd..

[9] Gallardo MJ, Supnet RA, Ahmed K (2018). Viscodilation of Schlemm's canal for the reduction of IOP via an ab-interno approach. Clin. Ophthalmol..

[10] Riva I, Brusini P, Oddone F, Michelessi M, Weinreb RN, Quaranta L (2019). Canaloplasty in the treatment of open-angle glaucoma: A review of patient selection and outcomes. Adv. Ther..

[11] Bull H, von Wolff K, Körber N, Tetz M (2011). Three-year canaloplasty outcomes for the treatment of open-angle glaucoma: European study results. Graefes Arch. Clin. Exp. Ophthalmol..

[12] Mastropasqua L (2012). In vivo analysis of conjunctiva in canaloplasty for glaucoma. Br. J. Ophthalmol..

[13] Klink T (2012). Are there filtering blebs after canaloplasty?. J. Glaucoma..

[14] Lewis RA (2011). Canaloplasty: Three-year results of circumferential viscodilation and tensioning of Schlemm canal using a microcatheter to treat open-angle glaucoma. J. Cataract Refract. Surg..

[15] Vaziri K (2015). Incidence of bleb-associated endophthalmitis in the United States. Clin. Ophthalmol..

[16] Rashaed SA (2016). Endophthalmitis trends and outcomes following glaucoma surgery at a tertiary eye care hospital in Saudi Arabia. J. Glaucoma..

[17] Grieshaber MC, Pienaar A, Stegmann R (2019). Flap-sparing canaloplasty: A modified approach to Schlemm’s canal. Graefes Arch. Clin. Exp. Ophthalmol..

[18] Grieshaber MC, Schoetzau A, Flammer J, Orgül S (2013). Postoperative microhyphema as a positive prognostic indicator in canaloplasty. Acta Ophthalmol..

[19] Harvey BJ, Khaimi MA (2011). A review of canaloplasty. Saudi J. Ophthalmol..

[20] Rękas M, Petz K, Wierzbowska J, Byszewska A, Jünemann A (2014). Evacuating a pre-descemet hematoma through a clear corneal incision during a canaloplasty procedure. J. Cataract Refract. Surg..

[21] Freiberg FJ, Salgado JP, Grehn F, Klink T (2012). Intracorneal hematoma after canaloplasty and clear cornea phacoemulsification: Surgical management. Eur. J. Ophthalmol..

[22] Brusini P, Tosoni C (2012). Canaloplasty after failed trabeculectomy: A possible option. J. Glaucoma..

[23] Davids AM (2019). Ab interno canaloplasty (ABiC)-12-month results of a new minimally invasive glaucoma surgery (MIGS). Graefes Arch. Clin. Exp. Ophthalmol..

[24] Konopińska J (2015). Prospective randomized study comparing combined phaco-ExPress and phacotrabeculectomy in open angle glaucoma treatment: 12-month follow-up. J. Ophthalmol..

[25] Stawowski Ł (2015). Comparison of ExPress mini-device implantation alone or combined with phacoemulsification for the treatment of open-Angle Glaucoma. J. Ophthalmol..

[26] Rękas M, Barchan-Kucia K, Konopińska J, Mariak Z, Żarnowski T (2015). Analysis and modeling of anatomical changes of the anterior segment of the eye after c-ataract surgery with consideration of different phenotypes of eye structure. Curr. Eye. Res..

[27] Byszewska A, Konopińska J, Kicińska AK, Mariak Z, Rękas M (2019). Canaloplasty in the treatment of primary open-angle glaucoma: Patient selection and perspectives. Clin. Ophthalmol..

