# Supplementary material for: Mini-canaloplasty as a modified technique for the surgical treatment of open-angle glaucoma
Source: Sci Rep. 2020 Jul 30;10:12801. doi: 10.1038/s41598-020-69261-y (PMC7393495; doi:10.1038/s41598-020-69261-y)
Supplement: Supplementary file 1 — Supplementary Information. [file 41598_2020_69261_MOESM1_ESM.docx]

Supplementary information

Supplementary Video S1. Mini-canaloplasty is a modified technique of glaucoma surgery.

Legend: Mini-canaloplasty without creating intrascleral lake
